# Anti-lipoapoptotic effects of Alisma orientalis extract on non-esterified fatty acid-induced HepG2 cells

**DOI:** 10.1186/s12906-016-1181-2

**Published:** 2016-07-25

**Authors:** Hyeon-Soo Jeong, Young-Hwan Cho, Kang-Hoon Kim, Yumi Kim, Ki-Suk Kim, Yun-Cheol Na, Jiyoung Park, In-Seung Lee, Jang-Hoon Lee, Hyeung-Jin Jang

**Affiliations:** 1 College of Korean Medicine, Institute of Korean Medicine, Kyung Hee University, 1 Heogi-dong, Dongdaemun-gu, Seoul 130-701 Republic of Korea; 2 Western Seoul Center, Korea Basic Science Institute, 150 Bugahyeon-ro, Seodaemun-gu, Seoul 120-140 Republic of Korea

**Keywords:** *Alisma orientale*, HepG2, Nonalcoholic fatty liver disease, De novo lipogenesis, Lipoapoptosis, Inflammation

## Abstract

**Background:**

Liver steatosis was caused by lipid accumulation in the liver. *Alisma orientale* (AO) is recognized as a promising candidate with therapeutic efficacy for the treatment of nonalcoholic fatty liver disease (NAFLD). HepG2 hepatocyte cell line is commonly used for liver disease cell model.

**Method:**

The HepG2 cells were cultured with the NEFAs mixture (oleic and palmitic acids, 2:1 ratio) for 24 h to induce hepatic steatosis. Then different doses of *Alisma orientale* extract (AOE) was treated to HepG2 for 24 h. Incubated cells were used for further experiments.

**Results:**

The AOE showed inhibitory effects on lipid accumulation in the Oil Red O staining and Nile red staining tests with no cytotoxicity at a concentration of 300 μg/mL. Fatty acid synthase (FASN) and acetyl-CoA carboxylase 1 (ACC1) mRNA and protein expression level were down-regulated after AOE treatment. Bcl-2 associated X protein (Bax) and c-Jun N-terminal kinase (JNK) mRNA expression level were decreased as well as p-JNK (activated form of JNK), Bax, cleaved caspase-9, caspase-3 protein expression level. Anti-apopototic B-cell lymphoma 2 (Bcl-2) protein level increased after AOE treatment. In addition, inflammatory protein expression including p-p65, p65, COX-2 and iNOS were inhibited by AOE treatment.

**Conclusion:**

The results suggest that AOE has anti-steatosis effects that involve lipogenesis, anti-lipoapoptosis, and anti-inflammation in the NEFA-induced NAFLD pathological cell model.

## Background

Hepatic steatosis has been defined chemically as an intrahepatic triglyceride (TG) content that is more than 5 % of the liver volume or weight, and histologically as more than 5 % of the hepatocytes containing visible intracellular TG [1, 2]. Nonalcoholic fatty liver disease (NAFLD) stands for a spectrum of diseases ranging from simple steatosis to steatohepatitis through to fibrosis and cirrhosis [3]. Although the prevalence of NAFLD varied between different regions an countries, the accepted global prevalence is 20–30 % for NAFLD and 2–3 % for nonalcoholic steatohepatitis (NASH) [4]. In Korea, according to a study using sonography surveys, it is estimated that 28.1 % of the Korean nondiabetic population suffers from NAFLD [5]. The reported risk factors for NAFLD include obesity, insulin resistance, hyperlipidemia, and hyperglycemia [6] and the increasing prevalence of obesity makes NAFLD a potential emerging pandemic [7].

Lipoapoptosis is a term representing a pathogenic phenomenon induced when non-esterified fatty acids (NEFAs) overwhelm the esterifying capacity of the liver. Excessive NEFAs may induce lipotoxicity including the promotion of apoptosis and, therefore, the phenomenon is termed lipoapoptosis [8, 9].

Hepatic steatosis develops when the rate of fatty acid (FA) input outweighs the output. de novo lipogenesis (DNL), one of the FA input components, is concerned with the FA synthesis mechanism of the liver [2]. And fatty acid synthase (FASN) and acetyl-CoA carboxylase 1 (ACC1) are the key genes associated with DNL [10].

Saturated NEFA causes cell death via sustained activation of the c-Jun N-terminal kinase (JNK). Through several complex processes, activated JNK activates Bcl-2-associated X protein (BAX), a multidomain proapoptotic member of the B-cell lymphoma 2 (Bcl-2) family, which then translocates to the mitochondria, causing its dysfunction and caspase-dependent cell death [11].

Nuclear factor kappa-light-chain-enhancer of activated B cells (NF-kB), which is a key transcription factor that regulates the genes related to the immune response and inflammation is activated by NEFA induction. Dysregulation of the NF-kB pathway plays an essential role in many diseases such as inflammatory diseases, cancer, and atherosclerosis [12, 13].

*Alisma orientale* (AO) has been known to have anti-inflammatory and diuretic effects in traditional medicine [14] and is prescribed to treat diseases including inhibited urination, edema, strangury disease, disease associated with fluid retention [15]. Several previous studies have reported the therapeutic efficacy of AO in NAFLD. The aqueous extract of AO showed anti-apoptotic and inhibitory effects against cellular steatosis and reactive oxygen species (ROS) production in a palmitate-induced cellular model [15, 16]. The 80 % ethanol extract of AO had been reported to show hepatocellular protective effects mediated by the regulation of apoptosis-related proteins [17].

In this study, additional attempt was made to reveal effects against pathological mechanisms including lipid accumulation, lipoapoptosis and inflammation induced by NEFA. HepG2 cells were treated with the 30 % ethanol *Alisma orientale* extract (AOE) and a 3-(4,5-dimethylthiazol-2-yl)-2,5-diphenyltetrazolium bromide (MTT) assay was performed to determine the safe concentration. In addition, Oil Red O staining and Nile red staining were performed to visually assess the effect of AOE against hepatocyte lipid accumulation. Furthermore, mRNA real-time quantitative polymerase chain reaction (qPCR) and western blot analysis were performed to evaluate NAFLD related factors including ACC1 and FASN markers including DNL, Bcl-2, BAX, JNK, caspase-3, and −9 and key molecules of inflammation associated with p-65, COX-2, and iNOS.

## Method

### Materials

Dulbecco’s modified Eagle’s medium (DMEM), and fetal bovine serum (FBS) were purchased from Lonza (Walkersville, MD, USA). Bovine serum albumin (BSA) was purchased from RMBIO (6015 Greg`s Way Missoula, MT, USA, Cat# : BSA-BLP-1XG). Oil Red O, oleic and palmitic acids (Cat# : O0625-25G), Nile red (Cat# : N3013-100MG), and the MTT (Cat# : M2003-1G) were purchased from Sigma-Aldrich (Saint Louis, MO, USA). The total RNA isolation and cDNA synthesis kits were purchased from GeneAll Bioscience (Seoul, Korea, Cat# : 305–101). The SYBR green master mix was purchased from Life Technologies (Carlsbad, CA, USA, Cat# : 43676569). BAX (Cat# : ab10813) and Bcl-2 (Cat# : ab59348) antibodies were purchased from Abcam (Cambridge, UK). JNK (Cat# : 9252), p-JNK (Cat# : 9251), caspase-9 (Cat# : 9502), cleaved caspase-9 (Cat# : 9501), caspase-3 (Cat# : 9665), and cleaved caspase-3 (Cat# : 9661) antibodies were purchased from Cell Signaling Technology (Danvers, MA, USA). β-actin (Cat# : sc-47778) and FASN (Cat# : 55580) antibodies were purchased from Santa Cruz Biotechnology (Santa Cruz, CA, USA). The enhanced chemiluminescence (ECL) solution (Luminata™ crescendo western horseradish peroxidase, HRP substrate, Cat# : WBLUR0100) was purchased from Merck Millipore (Jeffrey, NH, USA).

### Herbal extraction

The *Alisma orientale* was obtained from the Kyung Hee Oriental Herbal Medicine Research Center (Seoul, Korea) [18]. *Alisma orientale* was purchased by Omniherb (Yeoncheon, South Korea), which had sale as a nationally authorized distribution enterprise of herbal medicine. The product number of *Alisma orientale* was #: DH1602025041K. The preparation of the samples is described in the cited references excluding the solvent composition [19, 20]. *Alisma orientale* was ground to the appropriate particle size and extracted in 30 % ethanol. Then, ultrasound-assisted extraction was performed twice at 40 °C for 3 h. The extracted *Alisma orientale* sample was filtered, and continuously evaporated using a rotary evaporator. The dried *Alisma orientale* extracts powder was used in this experiment.

### Cell culture

HepG2 cells were obtained from the Korea Cell Line Bank (Seoul, Korea). The cells were maintained in DMEM supplement with 10 % FBS, 1 % antibiotics and antimycotic (ABAM) solution, and then incubated at 37 °C in a 5 % CO_2_ humidified atmosphere [21]. For the experiments, the cells were seeded in a 24-well cell culture plate at a density of 5 × 10^5^ cells per well and incubated for 16 h, and then washed with PBS and incubated with low glucose DMEM for 12 h for serum starvation. Then, the medium was changed to DMEM with 0.5 mM NEFA (0.33 mM oleic acid and 0.17 mM palmitic acid) (with 1 % BSA), and the cells were incubated further for 24 h to induce the hepatosteatotic condition. Then, the medium was changed to DMEM containing 10 % FBS, 1 % ABAM, and appropriate doses of the drugs and cells were incubated further for 24 h.

### Cell viability assay

Cell viability was measured using the MTT assay. The cells were seeded (10 × 10^3^ cells/well) in a 96-well cell culture plate, incubated for 48 h, and then treated with different concentrations of AOE (1, 10, 50, 100, 300, 500, 1000 μg/mL) for 24 h. The MTT was dissolved at 1 mg/mL in phosphate-buffered saline (PBS). After incubation, the medium was discarded, and the cells were treated with 100 μL of MTT solution for 1 h. Then the solution was removed, and dimethyl sulfoxide (DMSO) was added to dissolve the insoluble formazan crystal, which were measured at an absorbance of 570 nm using a Bio-Rad model 680 microplate reader [22].

### Oil red O staining

The steatosis-induced HepG2 cells were washed with ice-cold PBS to remove unbound stain, and then fixed in ice-cold 10 % formalin for 30 min. The cells were then washed with distilled water, and stained for 30 min with 0.3 % Oil Red O solution (in 60 % isopropanol) at room temperature for the stain lipid droplets to form. The stained cells were washed with running tap water for 2 min, and immediately counterstained with Harris’s hematoxylin before cell drying. The cells were observed and photographed using an optical microscope (Olympus IX71).

### Nile red staining

The cells were washed with ice-cold PBS, fixed for 30 min in ice-cold 10 % formalin, washed with PBS, then stained with 0.3 nM Nile red solution (in PBS) for 15 min in a dark room. After staining, the Nile red solution was removed, and the cells were washed with PBS, suspended in PBS, and collected in a polystyrene flow cytometry sample tube. The stained cell lipid was determined by measuring the fluorescence using the FACScalibur flow cytometer (BD Bioscience) and analyzed using the Cellquestpro (BD Bioscience).

### Real-time quantitative PCR

Total RNA was isolated from HepG2 cells using a RiboExtm kit (Geneall). A quantified 1-μg sample of total RNA was used to synthesize cDNA using a Legene 1st strand Express cDNA synthesis system kit (Legene Bioscience). The mRNA expression level was determined via qPCR according to the manufacturer’s manual. Among the results, the 2-ΔΔCT values were used to compare the groups and glyceraldehyde 3-phosphate dehydrogenase (GAPDH) was used as the endogenous control [23]. The sequences of the gene mRNA primers are shown in Table 1.

**Table 1:** List of real time PCR primer sequence

Gene	Forward primer	Reverse primer
*GAPDH*	GCCAC ATCGC TCAGA CACC	CCCAA TACGA CCAAA TCCGT
*BAX*	CACCA AGGTG CCGGA ACT	TCCCG GAGGA AGTCC AATG
*BCL-2*	CATGT GTGTG GAGAG CGTCA A	GCCGG TTCAG GTACT CAGTC A
*MAPK8*	TGGTC AGCAG GGTGT CACA	TCGCA GAGGG AGAAA AGCAA
*CASP9*	GGCTT ACATC CTGAG CATGG A	CGGCA GAAGT TCACA TTGTT GA
*FASN*	CGCTC GGCAT GGCTA TCT	CTCGT TGAAG AACGC ATCCA
*ACC1*	GGATG GTGTT CACTC GGTAA TAGA	GGGTG ATATG TGCTG CGTCA T

### Western blotting

The cells were washed, scraped into ice-cold PBS, and then incubated in radioimmunoprecipitation (RIPA) assay buffer with NP-40 and protease inhibitor cocktail for 1 h on ice for cell lysis. After lysis, a Bradford assay was performed to ensure the cell lysates contained the same protein quantities. Each protein (10 μg samples) was separated according to size using 10 % sodium dodecyl sulfate (SDS)-polyacrylamide gel electrophoresis (PAGE), and then transferred to a polyvinylidene fluoride (PVDF) membrane. The membrane was incubated with primary antibodies diluted 1:3000 in Tris-buffered saline-Tween (TBS-T) and the secondary antibody (1:10000 in TBS-T) in that order [24]. The protein blot was detected with the ECL solution using a Davinci-chemi chemiluminescence system (Davinch-K). The intensity of the protein blot was measured and calculated using the ImageJ software (NIH).

### Identification of AO30 compound

The analysis was performed in the Korea Basic Science Institute (Seoul, Korea). The system consisted of an Infinity 1290 ultra-performance liquid chromatography (UPLC) system and an electrospray ionization (ESI) quadruple time-of-flight (QTOF) mass spectrometer (MaS) (G6550A, Agilent, CA, USA). A supelco ascientis express c18 column (1.7 μm, 150 mm × 2.1 mm i.d.) was used as an analytical column. The column was maintained at 40 °C. The total running time, including the conditioning of the column to the initial conditions, was 50 min. The injection volume was 1 μL. The ESI-QTOF-MaS instrument was operated in the positive and negative ion mode using a dual agilent jetstream electrospray ionization source. The desolvation gas was set to a flow rate of 720 L/h at a temperature of 350 °C. The source temperature was set to 100 °C. The capillary voltage was set to 4000 V.

### Statistical analysis

All data represent at least two separate experiments performed in triplicate. The significance of the data was analyzed using the Prism 5 software with a one-way analysis of variance (ANOVA), Bonferroni’s post hoc test, and Student’s *t*-test to compare each set of data. Bars show the means ± standard error of the means (SEM). Significance was denoted as follows, ^*^p < 0.05, ^**^p < 0.01, and ^***^p < 0.001.

## Results

### Effects of AOE on HepG2 cell viability

**Fig. 1:**
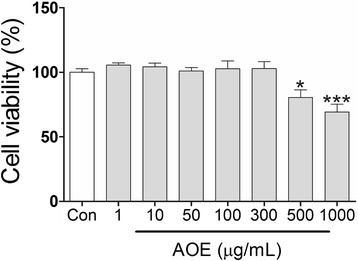
Cell viability assay showing cytotoxicity of *Alisma orientale* extract (AOE) in HepG2 cells. HepG2 cells were treated with different concentration of AOE for 24 h, and then MTT assay was performed to measure cell viability. Values are mean ± SEM. Statistical significant was determined using one-way ANOVA test, ^*^ *p* < 0.05 compared to control; ^***^ *p* < 0.001

*Alisma orientale* extracts (AOE) has clinically used to prescribe main component on inflammatory disease in Korean medicine. Even if clinical trials treated with AOE on prescription had not found, safe evaluation should need to research on experimental human hepatic system. To determine the cytotoxicity of AOE on human hepatic cell line, we performed MTT assay on HepG2 cell line (Fig. 1). Treatment of HepG2 cells with different concentrations (1–300 μg/mL) of AOE for 24 h did not significantly decrease the cell viability compared to the control group. On the other hand, high doses of AOE (500 and 1000 μg/mL) significantly decreased cell viability.

### Effects of AOE and NEFA on HepG2 cell lipid accumulation

**Fig. 2:**
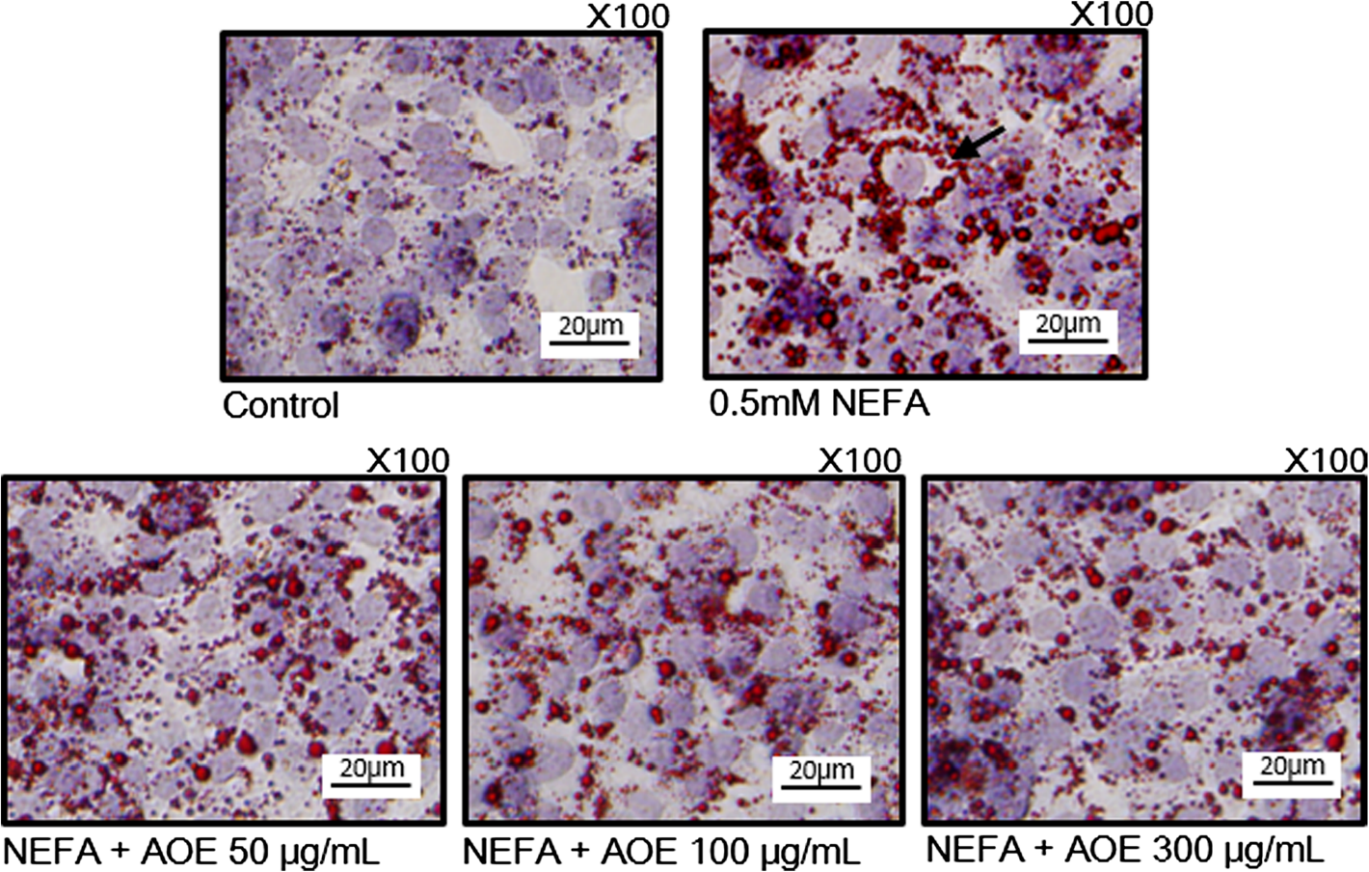
Oil Red O staining of HepG2 Cells. Inhibitory effect of AOE on lipid accumulation in HepG2 cell line. Red dot (arrowhead) shows lipid droplet; pale blue circle shows cell nucleus. Cells were starved for 12 h, then treated with 0.5 mM NEFA for 24 h, and then AOE for 24 h. Cells were observed under magnification of 100×, scale bar, 20 μm. AOE, *Alisma orientale* extract; NEFA, non-esterified fatty acid

**Fig. 3:**
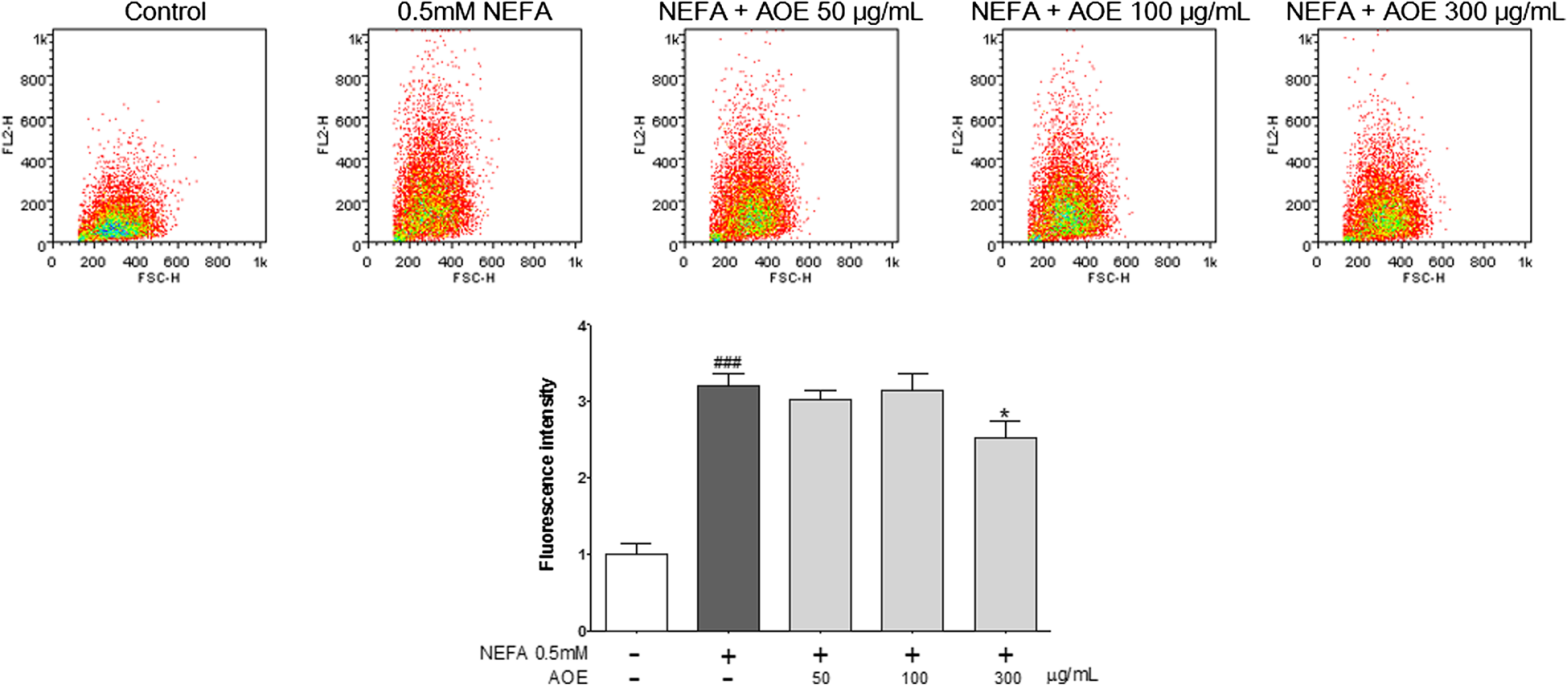
Nile red staining of HepG2 Cells and quantitative analysis of lipids. Inhibitory effect of AOE on lipid accumulation in HepG2 cell line. Nile red fluorescence was measured using flow cytometry. Fluorescence dot plot and relative fluorescence graph. Values are mean ± SEM, ^###^ *p* < 0.001, compared to control, ^*^ *p* < 0.05, compared to NEFA-treated group. AOE, *Alisma orientale* extract; NEFA, non-esterified fatty acid

To determine if AOE decreased the hepatocyte lipid accumulation in NEFA-induced steatotic conditioned HepG2 cells [25], Oil Red O staining and Nile red staining were performed. In the Oil Red O staining (Fig. 2), the HepG2 cells accumulated lipid droplets were stained red. Compared with the control, the NEFA-treated HepG2 cells showed an increase in cell lipid accumulation (arrows). In addition, after the AOE treatment, the cell lipid decreased dose-dependently (50, 100, and 300 μg/mL). Also, to quantify the cell lipid content, the Nile red staining assay was performed, and the fluorescence count was determined using flow cytometry (Fig. 3). The relative fluorescence intensity (Fig. 3) shows that cell lipid droplets decreased following AOE treatment. However, the effect was only significant at a concentration of 300 but not of 100 μg/mL.

### Effects of AOE on HepG2 cell lipogenesis related mRNA and protein expression

**Fig. 4:**
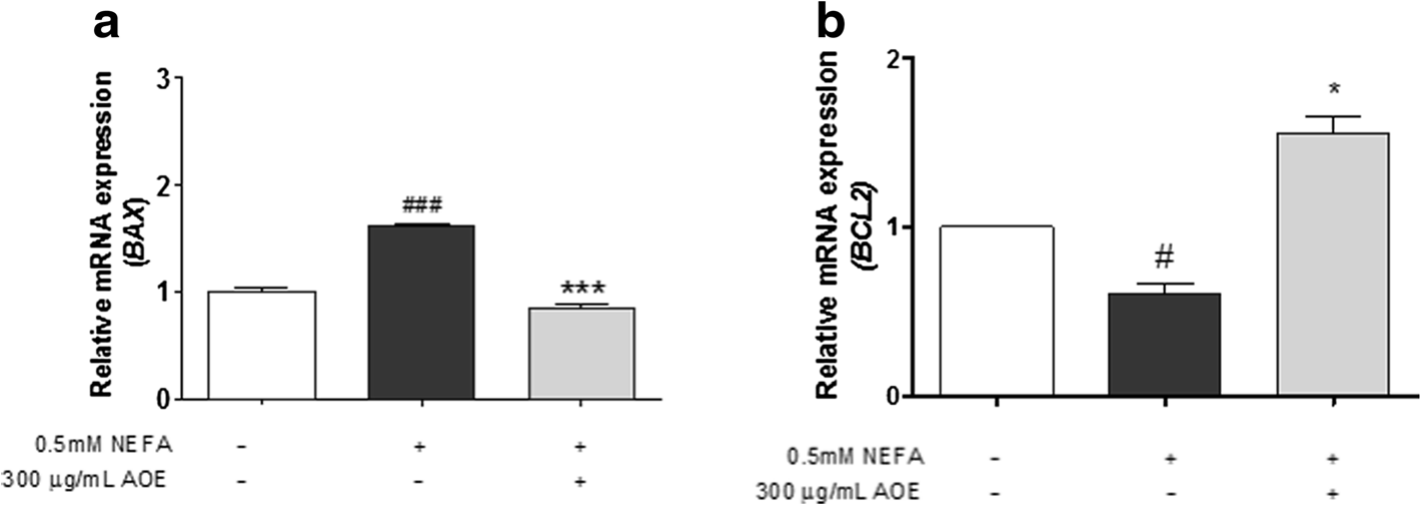
Bcl-2-associated X protein (BAX), and B-cell lymphoma 2 (Bcl-2) mRNA expression levels. Inhibitory effect of AOE on lipogenesis-related mRNA expression in HepG2 cells. mRNA expression level was measured using SYBR green qPCR. Cells were starved for 16 h, treated with 0.5 mM NEFA for 24 h, and then 300 μg/mL AOE for 24 h. Total RNA was then extracted and reverse transcribed using PCR and qPCR. Fatty acid synthesis-related **a**, **b** gene expression level was calculated using *GAPDH* expression level as an internal control. Statistical significancy was determined using Student’s *t*-test. Values are mean ± SEM, ^##^ *p* < 0.01 compared to control, ^###^ *p* < 0.001, ^***^ *p* < 0.01 compared to NEFA-treated group. AOE, *Alisma orientale* extract; NEFA, non-esterified fatty acid; qPCR, real-time polymerase chain reaction; *GAPDH*, glyceraldehyde 3-phosphate dehydrogenase

To reveal the pathway involved in the AOE-mediated decrease in hepatocyte lipid accumulation, a qPCR was performed. FASN and ACC1 protein are related to the cell lipid accumulation and lipogenesis induced by NEFA [26]. Compared with control, the NEFA-treated group showed a significantly increased mRNA expression. In addition, the AOE-treated group showed significant inhibition of FASN and ACC1 expressions (Fig. 4a and b).

### Down-regulation of cell lipoapoptosis-related mRNA and protein expression by AOE in HepG2 cells

**Fig. 5:**
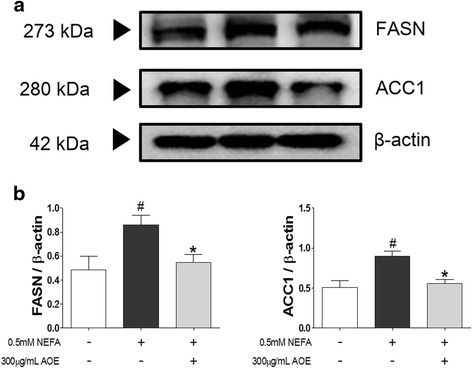
Fatty acid synthase (FASN) and acetyl-CoA carboxylase 1 (ACC1) protein expression levels. Lipogenesis-related protein regulatory effects of AOE in HepG2 cells. Cell lysates were obtained using NP40 lysis buffer and 15 μg of protein was loaded on a 12 % SDS-PAGE gel. After separation, proteins were transferred to PVDF membrane. **a** Western blots were attached by anti-FASN or anti-ACC1 respectively and their attached proteins were detected using Davinci-K chemiluminescence detector. **b** The dectected proteins of FASN or ACC1 were described by bar graph. Western blot was performed in triplicate. β-actin protein was used as an internal control. Statistical significant was determined using Student’s *t*-test. Values are mean ± SEM, ^#^ *p* < 0.05 compared to control, ^*^ *p* < 0.05 compared to NEFA-treated group. AOE, *Alisma orientale* extract; NEFA, non-esterified fatty acid; SDS-PAGE, sodium dodecyl sulfate-polyacrylamide gel electrophoresis

**Fig. 6:**
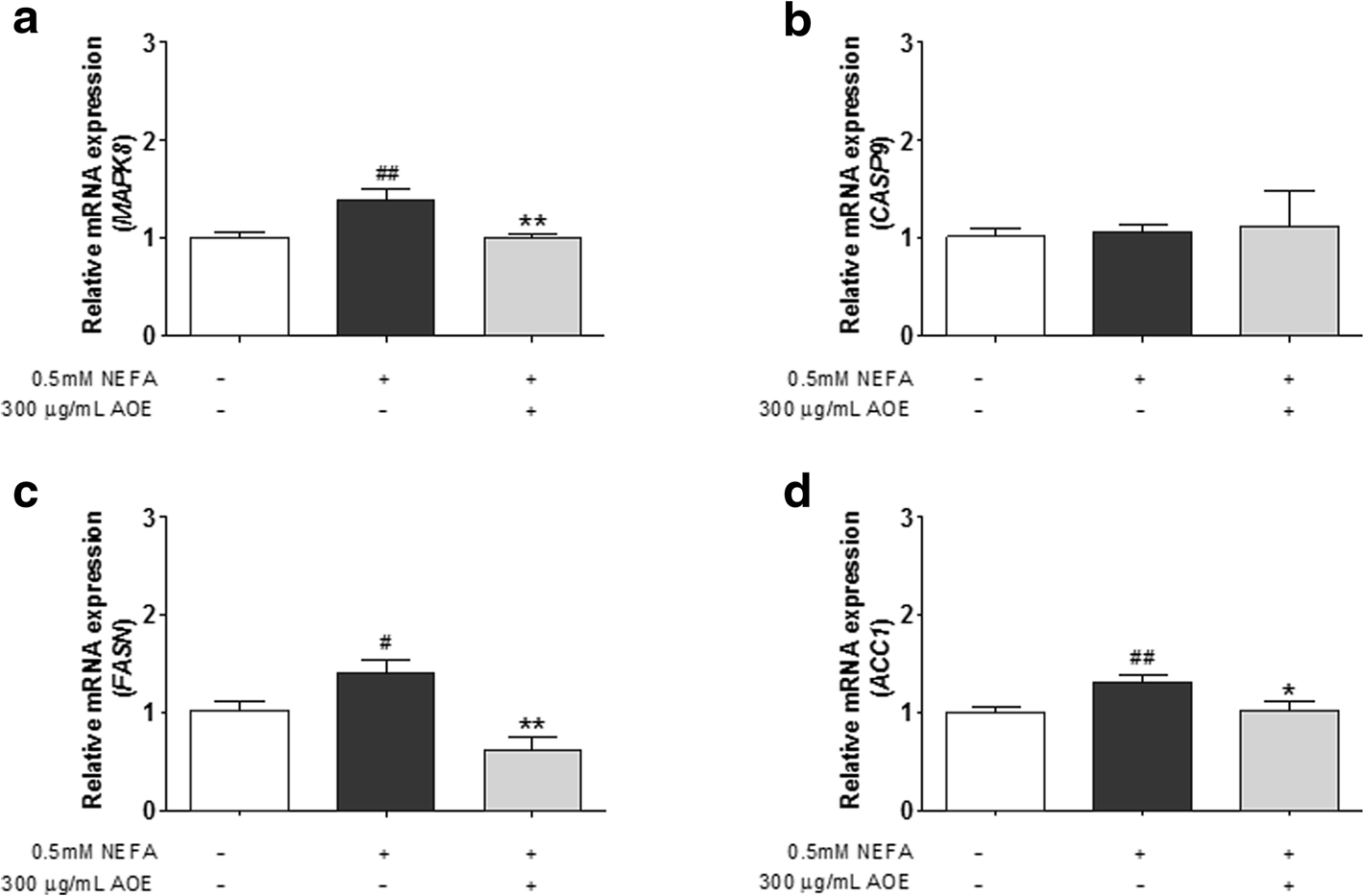
Mitogen-activated protein kinase (MAPK) 8, caspase-9 mRNA expression levels, Fatty acid synthase (FASN) and acetyl-CoA carboxylase 1 (ACC1). Inhibitory effect of AOE on lipoapoptosis-related mRNA expression in HepG2 cells. The mRNA expression level was measured using SYBR green qPCR. Cells were starved 16 h, treated with 0.5 mM NEFA for 24 h, and then with 300 μg/mL AOE for 24 h. Total RNA was then extracted reverse transcribed using PCR and qPCR. **a**-**d**, Lipoapoptosis-related gene expression level was calculated using *GAPDH* expression level as an internal control. Statistical significant was determined using Student’s *t*-test. Values are mean ± SEM, ^#^ *p* < 0.05 compared to control, ^##^ *p* < 0.01, ^*^ *p* < 0.05 compared to NEFA-treated group, ^**^ *p* < 0.01. qPCR, real-time polymerase chain reaction; AOE, *Alisma orientale* extract; NEFA, non-esterified fatty acid; *GAPDH*, glyceraldehyde 3-phosphate dehydrogenase

**Fig. 7:**
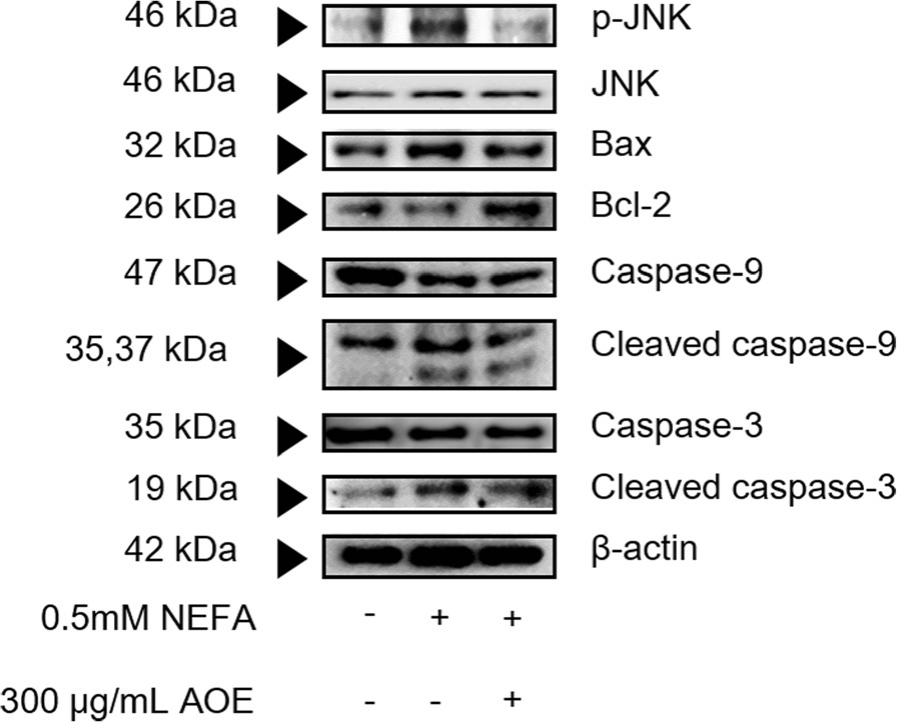
Lipoapoptosis-related protein expression levels. Apoptosis-related protein regulatory effects of AOE in HepG2 cells. Cell lysates were obtained using NP40 lysis buffer, and 15 μg of protein was loaded on a 12 % SDS-PAGE gel. After separation, proteins were transferred onto a PVDF membrane. Protein blot was detected using a Davinci-K chemiluminescence detector. β-actin protein was used as an internal control. AOE, *Alisma orientale* extract; SDS-PAGE, sodium dodecyl sulfate-polyacrylamide gel electrophoresis

FASN and ACC1 protein expressions (Fig. 5a) were increased in the NEFA-treated group compared to the control group. In addition, the AOE-treated group showed down-regulation of FASN and ACC1 protein expression compared to the NEFA-treated group (Fig. 5a). In addition, the protein expression intensity (Fig. 5b) of FASN and ACC1 in the AOE-treated group was more significantly decreased than the group treated with NEFA. JNK protein causes potential cellular damage via the lipoapoptosis pathway following its activation by NEFA accumulation in cells. NEFA also increases beta-oxidation in the cell mitochondria and induces the release of cytochrome C, thereby triggering the lipoapoptosis pathway [27, 28]. Compared with the control, the NEFA-treated group showed a significant increase in the mRNA expression of BAX, Bcl-2, and mitogen-activated protein kinase (MAPK) 8 (JNK mRNA, Fig. 6a, b, and c) but not caspase-9 (Fig. 6d). In addition, the AOE-treated group showed a significant inhibition of BAX and MAPK8 (Fig. 6a and c) but no significant change in Bcl-2 protein expressions. Phosphorylated (p)-JNK protein expression (Fig. 7) was increased when HepG2 cells were treated NEFA and this effect was decreased following AOE treatment. Other cell apoptosis-related proteins including BAX showed increased expression in the NEFA-treated group while Bcl-2 protein expression was suppressed. In addition, the AOE-treated cells showed not only down-regulation of BAX expression, but also up-regulation of Bcl-2 expression. Caspase-3 is activated by caspase-9 [24–29], and the levels of both cleaved forms increased in NEFA-treated cells and decreased following AOE treatment.

### Effects of AOE on inflammatory protein expression in HepG2 cells

**Fig. 8:**
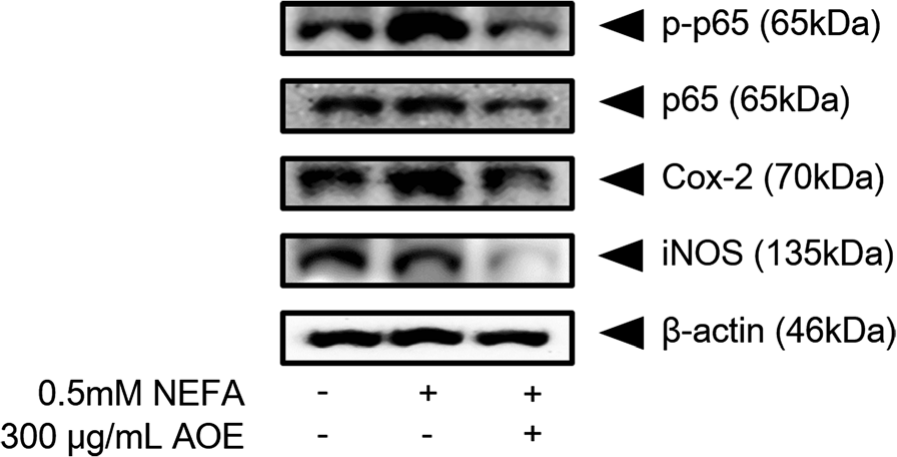
Inflammatory protein expression levels. Inflammatory protein regulatory effect of AOE in HepG2 cells. Cell lysates were obtained using NP40 lysis buffer and 15 μg of protein was loaded on a 12 % SDS-PAGE gel. After separation, proteins were transferred to a PVDF membrane. Protein blot was detected using a Davinci-K chemiluminescence detector. β-actin protein was used as an internal control. AOE, *Alisma orientale* extract; SDS-PAGE, sodium dodecyl sulfate-polyacrylamide gel electrophoresis

The NF-kB p65 (p65) is a well-known cell inflammatory protein, which can be activated by NEFA [13]. Among the result of inflammatory protein expression (Fig. 8), p-p65 and COX-2 expression was highly increased compared to the control, and the AOE-treated group showed a decrease in p-p65 and COX-2 protein expression. In addition, p65 and inducible nitric oxide synthase (iNOS) protein expression was not different between the NEFA-treated and control groups but the AOE-treated group showed a decreased in both protein expressions.

### High performance liquid chromatography (HPLC) of Alisma orientale extracts

**Fig. 9:**
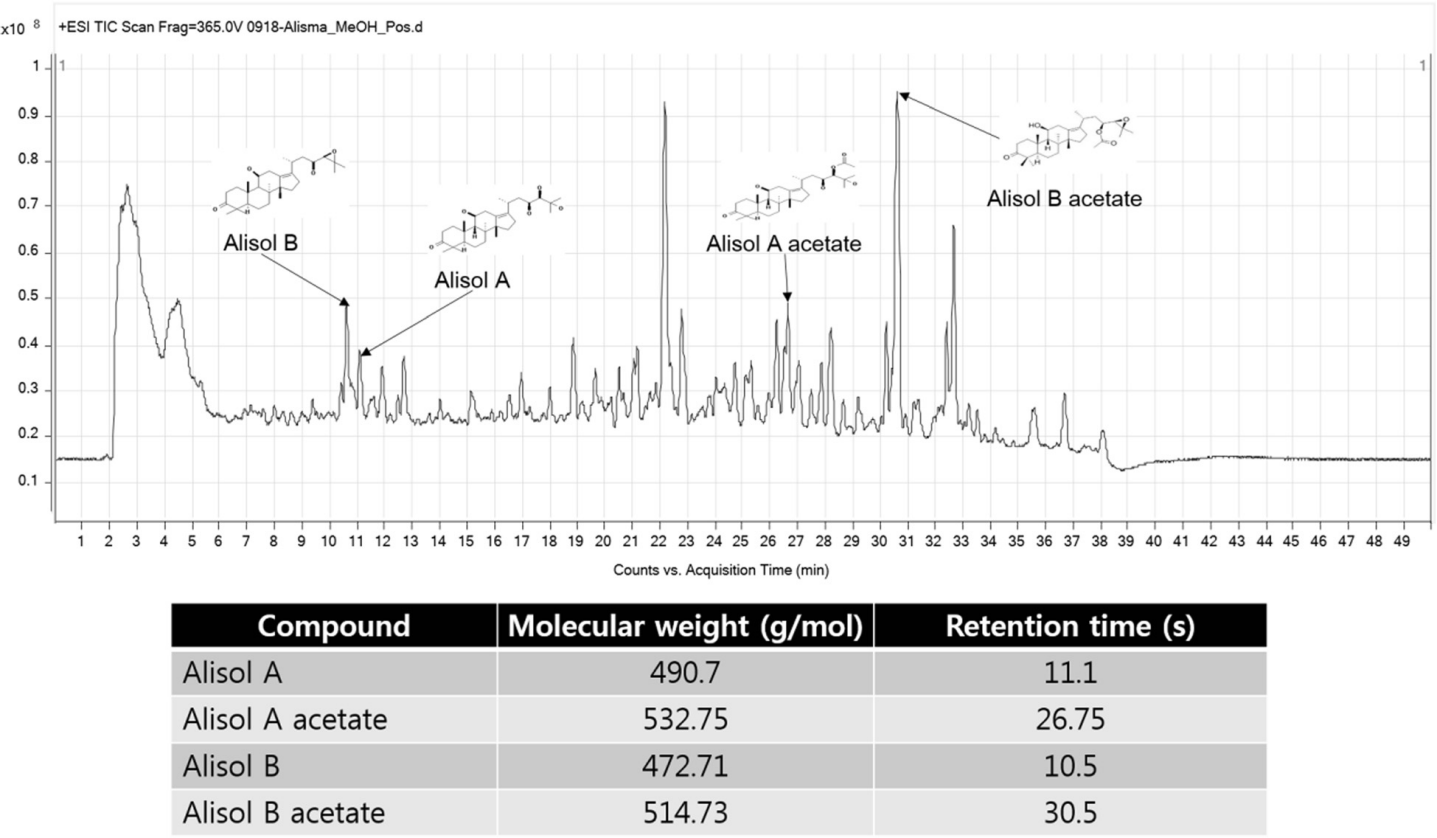
AO30 high performance liquid chromatography. AO30 sample was diluted in 30 % ethanol; the vehicle liquid contained distilled water and acetonitrile. AO30 HPLC results showed that AO30 has alisol A, alisal A acetate, alisol B, and alisol B acetate

Alisol A, alisol A acetate, alisol B, and alisol B acetate were the key ingredients of AO. We investigated whether the experimental extract on AOE has the key ingredients by using high performance liquid chromatography (HPLC). The chromatogram result showed that AOE has alisol A, alisal A acetate, alisol B, and alisol B acetate (Fig. 9).

## Discussion

This study was designed to investigate the anti-steatotic effects of AOE using NEFA-induced HepG2 cells as lipogenesis markers that contribute to lipid accumulation. In addition, the anti-lipoapoptotic and anti-inflammatory effects of AOE were also investigated in the same cell line model. To determine the cytotoxicity of AOE, the MTT assay was performed. In addition, the Oil Red O staining and Nile red staining were used to determine the inhibitory effects of AOE on hepatocyte lipid accumulation. Furthermore, we sought to determine if the pathway mediating the effects of AOE involves the reduction of hepatocyte lipid accumulation, lipoapoptosis, and inflammation as they relate to the progression from simple steatosis to NASH. NASH is characterized histologically by hepatic steatosis with evidence of liver cell inflammation and injury (ballooning degeneration) [30]. Although the mechanisms underlying the progression from steatosis to NASH is still not fully elucidated, increasing evidence suggests that oxidative stress, which occurs when ROS are generated to certain levels and not appropriately removed, is a key driving force in triggering inflammatory signaling pathways that promote the progression of simple steatosis to NASH [26–32]. Therefore, mRNA qPCR and western blot were performed to evaluate the expression of the specific mRNA and proteins involved in these phenomena.

The MTT assay was used to determine the AOE concentrations that safe for use in this study. Concentration of AOE was up to 300 μg/mL showed no significant decrease in the viability of the treated HepG2 cells compared to the control group. However, higher doses of AOE (500 and 1000 μg/mL) significantly decreased the cell viability (Fig. 1). In previous studies using the 80 % ethanol AOE, 100 μg/mL had no significant cytotoxicity while concentrations of 500 and 1000 μg/mL of AOE caused a significant decrease in cell viability [17], similar to our result. Based on this result, a concentration of 300 μg/mL of AOE could be used in future experiments.

FAs are classified chemically as saturated and unsaturated (monounsaturated and polyunsaturated), and their structures affect their biological activity [33]. Saturated fatty acids (SFAs) are known to induce lipotoxicity such as insulin resistance and cell death. Palmitic and oleic acids are a typical SFA and monounsaturated fatty acid (MUFA), respectively [34]. In a study of the differential effects of these FAs on apoptosis and lipid accumulation in cultured hepatocytes, oleic acid was revealed to be more steatogenic but less apoptotic than palmitic acid was. Incubation with both FAs resulted in a higher amount of fat accumulation than with palmitic acid alone, but the apoptosis was lower, indicating a protective feature for oleic acid [33]. A proper ratio of palmitic and oleic acids in a combination mixture leads to significant lipoapoptosis and intracellular lipid accumulation with minimal cellular damage [18, 19]. In one study, NEFAs composed of oleic and palmitic acids (2:1), maximized lipid accumulation without causing severe cellular toxicity [35] and a 0.5 mM mixture of NEFAs in the same ratio showed sufficient lipid accumulation in HepG2 cell in the Oil Red O and Nile red staining tests. Based on these results, the study design used a combination NEFA consisting of oleic acid and palmitic acid in a 2:1 molar ratio for developing the pathological cell model of NAFLD.

In the Oil Red O staining test, AOE reduced the 0.5 mM NEFA-induced cell lipid accumulation dose-dependently up to a concentration of 300 μg/mL (Fig. 2). The Nile red staining was performed using flow cytometry to quantify the cell lipid accumulation, and the relative fluorescence intensity showed that the cell lipid decreased significantly following treatment with 300 but not 50 or 100 μg/mL concentrations of AOE (Fig. 3). This result suggests that 300 μg/mL of AOE has an effect on lipid accumulation in HepG2 cells without any cell toxicity.

The possible sources of the fats that are responsible for hepatic steatosis are peripheral fats contained in adipose tissue, which flow to the liver via the plasma NEFA pool, FAs newly formed in the liver by DNL, and dietary FAs. DNL is an endogenous pathway, which converts carbohydrates and proteins to NEFAs [2]. Furthermore, in the fasting state, roughly one-fourth of all the FAs are newly made via DNL [36]. FASN and ACC1 are key genes associated with DNL [10] and regulated by sterol regulatory element-binding protein (SREBP)-1c. ACC1 forms the malonyl-CoA that is used by FASN to synthesize palmitic acid, and it is possible to synthesize oleic acid by successive elongation and desaturation processes [32–37]. We investigated the inhibitory effects of AOE on the mechanism of DNL-mediated lipid accumulation using qPCR and western blotting to determine mRNA and protein levels. The NEFA-treated group showed a higher increased in the expression of FASN and ACC1 than the control group did. And the AOE-treated group showed higher down-regulation of FASN and ACC1 protein expression than the 0.5 mM NEFA-treated group did (Figs. 4 and 5). This result suggests that the effect of AOE on lipid accumulation is associated with the DNL process.

Factors related to lipoapoptosis, the pathological phenomenon induced by lipid accumulation were determined following the treatment of HepG2 cells with 0.5 mM NEFA and 300 μg/mL of AOE. When NEFAs penetrate cells, JNK is activated and the JNK protein, which is the central mediator of saturated NEFA induced hepatocyte apoptosis [38], and can causes potential cellular damage via the cellular apoptosis pathway [19, 28]. The JNK signaling pathway has been reported to affect members of the Bcl-2 family. Specifically, JNK can activate the mitochondrial translocation of BAX and inactivate anti-apoptotic Bcl-2 proteins [24–29]. BAX translocates to the mitochondria and thereby facilitates the release of the mitochondrial cytochrome C protein into the cytosol, which then interacts with procaspase-9, switching on caspase-3, to induce apoptosis [24–29, 34–39]. At the mRNA level, the AOE-treated group showed a more significant inhibition of BAX and MAPK8 expression but not caspase-9 than the 0.5 mM NEFA-treated group did. Protein expression levels of p-JNK (activated form of JNK), BAX, as well as cleaved caspase-9 and caspase-3 were more decreased in the AOE-treated HepG2 cells than they were in the 0.5 mM NEFA-treated cells, which corresponds to a previous study using 100 μg/mL of 80 % ethanol AOE and 1 mM mixed NEFAs in the same ratio [17]. The AOE treatment significantly affected Bcl-2 and caspase-9 protein expression levels (Figs. 6 and 7). Based on these result, the anti-lipoapoptosis was mediated via the JNK signaling pathway on HepG2 cell line treated with AOE.

NF-kB is known to play a critical role in the regulation of cell survival genes and coordination of pro-inflammatory mediators. NF-kB regulates the expression of iNOS, a pro-inflammatory enzyme that is involved in the production of nitric oxide (NO) at the transcriptional level [40, 41]. NF-kB also regulates COX-2, the key enzyme regulating the production of prostaglandins, which are central mediators of inflammation [42]. The p-p65 and COX-2 protein expression was more highly increased in the NEFA group than the control group. But, exposing the group treated with AOE showed remarkably decrease in their inflammatory protein expression. In summary, AOE was revealed to have anti-inflammatory effects in HepG2 cells.

From the result presented, AOE showed anti-steatosis effects mediate via lipogenesis, anti-lipoapoptosis, and anti-inflammatory mechanisms in the NEFA-induced NAFLD pathological cell model. We observed that AOE contributed not only to inhibiting simple steatosis, but also NASH based on the experiments involving lipoapoptosis and inflammation, which are factors involved in the progression of NAFLD. The described effects of AOE derived from Alisol A, Alisol acetate, Alisol B, and Alisol B acetate. Using the components of AOE, Alisol A, Alisol acetate, Alisol B, and Alisol B acetate need to discovery an individual pharmacological capacity in lipoapoptosis and anti-inflammation reageant.

## Conclusion

The anti-steatotic effects of AOE involving lipogenesis, anti-lipoapoptosis, and anti-inflammation in NEFA-induced HepG2 cells using the MTT assay, Oil Red O staining, Nile red staining, mRNA qPCR, and western blotting were investigated in this study. The summary of the results obtained is as follows,

1. AOE concentrations of up to 300 μg/mL caused no significant decrease in the viability of HepG2 cells compared to the control group.
2. AOE inhibited lipid accumulation in the Oil red O staining test and moreover, the Nile red staining test, which particularly enabled the quantification of cell lipid using flow cytometry, showed that cell lipid was decreased significantly following AOE treatment only at a concentration of 300 μg/mL.
3. AOE treatment down-regulated FASN and ACC1 protein expressions more than treatment with 0.5 mM NEFA did, and NEFA-treated cells showed a higher increase in the mRNA and protein expression levels of FASN and ACC1 than the control group cells.
4. AOE-treated HepG2 cells showed a significant inhibition of BAX and MAPK8 mRNA expression levels and of p-JNK (activated form of JNK) and BAX as well as cleaved caspase-9 and caspase-3 protein levels. Bcl-2 and caspase-9 protein but not mRNA expression levels were significantly affected by AOE treatment.
5. AOE- treated HepG2 cells showed decreased inflammatory protein expression including p-p65, COX-2, p65, and iNOS.
6. AOE was analyzed including in Alisol A, Alisol A acetate, Alisol B, Alisol B acetate by high performance liquid chromatography.

Based on these results, AOE is considered to have anti-steatosis effects, which involve lipogenesis, anti-lipoapoptosis, and anti-inflammation mechanisms in the NEFA-induced NAFLD pathological cell model. In the present study, AOE inhibited not only simple steatosis, but also the progression of the disease as evidenced by the results of the experiment involving lipoapoptosis and inflammation. Therefore, AO appears to be a potential candidate for development as a therapeutic option for the treatment of NAFLD, as well as NASH, which are induced by lipoapoptosis and inflammation.

## Abbreviations

ABAM, antibiotics and antimycotic; ACC, acetyl CoA carboxylase; ANOVA, one-way analysis of variance; AOE, *Alisma orientalis* extract; Bax, Bcl-2 associated X protein; Bcl-2, beta cell lymphoma 2; BSA, bovine serum albumin; COX-2, cyclooxygenase 2; DMEM, Dulbecco’s modified Eagle’s medium; DMSO, dimethylsulfoxide; DNL, de novo lipogenesis; ECL, enhanced ehmiluminescence; FASN, fatty acid synthase; FBS, fetal bovine serum; GAPDH, glyceraldehyde 3-phosphate dehydrogenase; HCC, hepatocellular carcinoma; iNOS, inducible nitric oxide synthase; MAPK, mitogen-activated protein kinase; MTT, 3-(4,5-dimethylthiazol-2-yl)-2,5-diphenyltetrazolium bromide; MUFA, monounsaturated fatty acid; NAFLD, non-alcoholic fatty liver disease; NASH, non-alcoholic steatohepatitis; NEFA, non-esterified fatty acid; NF-kB, nuclear factor kappa-light-chain-enhancer of activated B cells; NO, nitric oxide; PBS, phosphate buffer saline; p-JNK, phosphorylated jun-c-N-terminal kinase; PVDF, polyvinylidene fluoride; qPCR, quantitative polymerase chain reaction; RIPA, radioimmunoprecipitaion assay; ROS, reactive oxygen species; SDS, sodium dodecyl sulfate; SFA, saturated fatty acid; SREBP, sterol regulatory element-binding protein; TBS-T, tris-buffered saline-tween; TG, triglyceride

## References

[1] Bedogni G, Nobili V, Tiribelli C (2014). Epidemiology of fatty liver: An update. World Journal of Gastroenterology: WJG.

[2] Fabbrini E, Sullivan S, Klein S (2010). Obesity and nonalcoholic fatty liver disease: biochemical, metabolic, and clinical implications. Hepatology.

[3] Than NN, Newsome PN (2015). A concise review of non-alcoholic fatty liver disease. Atherosclerosis.

[4] Mario Masarone AF, Ludovico A, Carmela L, Marcello P (2014). Non Alcoholic Fatty Liver: Epidemiology and Natural History. Rev Recent Clin Trials.

[5] Bae JC, Cho YK, Lee WY, Seo HI, Rhee EJ, Park SE (2010). Impact of nonalcoholic fatty liver disease on insulin resistance in relation to HbA1c levels in nondiabetic subjects. Am J Gastroenterol.

[6] Park SH, Jeon WK, Kim SH, Kim HJ, Park DI, Cho YK (2006). Prevalence and risk factors of non-alcoholic fatty liver disease among Korean adults. J Gastroenterol Hepatol.

[7] Zambo V, Simon-Szabo L, Szelenyi P, Kereszturi E, Banhegyi G, Csala M (2013). Lipotoxicity in the liver. World J Hepatol.

[8] Cao J, Feng X-X, Yao L, Ning B, Yang Z-X, Fang D-L (2014). Saturated Free Fatty Acid Sodium Palmitate-Induced Lipoapoptosis by Targeting Glycogen Synthase Kinase-3β Activation in Human Liver Cells. Dig Dis Sci.

[9] Wang D, Wei Y, Pagliassotti MJ (2006). Saturated fatty acids promote endoplasmic reticulum stress and liver injury in rats with hepatic steatosis. Endocrinology.

[10] Auguet T, Berlanga A, Guiu-Jurado E, Martinez S, Porras JA, Aragonès G (2014). Altered Fatty Acid Metabolism-Related Gene Expression in Liver from Morbidly Obese Women with Non-Alcoholic Fatty Liver Disease. Int J Mol Sci.

[11] Kakisaka K, Cazanave SC, Fingas CD, Guicciardi ME, Bronk SF, Werneburg NW (2012). Mechanisms of lysophosphatidylcholine-induced hepatocyte lipoapoptosis. Am J Physiol Gastrointest Liver Physiol.

[12] Huang CY, Chen WM, Tsay YG, Hsieh SC, Lin Y, Lee WJ (2015). Differential regulation of protein expression in response to polyunsaturated fatty acids in the liver of apoE-knockout mice and in HepG2 cells. J Biomed Sci.

[13] Vinciguerra M, Veyrat-Durebex C, Moukil MA, Rubbia-Brandt L, Rohner-Jeanrenaud F, Foti M (2008). PTEN down-regulation by unsaturated fatty acids triggers hepatic steatosis via an NF-kappaBp65/mTOR-dependent mechanism. Gastroenterology.

[14] Hong X, Tang H, Wu L, Li L (2006). Protective effects of the Alisma orientalis extract on the experimental nonalcoholic fatty liver disease. J Pharm Pharmacol.

[15] Han CW, Joo MS, Lee JH (2012). Comparison of the Therapeutic Efficacy of Rhizoma Alismatis, Fructus Crataegi, Fructus Lycii, Radix Curcumae, Radix Salviae Miltiorrhizae, Herba Artemisiae Scopariae on the Experimental Cellular Model of Nonalcoholic Fatty Liver Disease. J Korean Orient Intern Med.

[16] Han CW, Kang ES, Ham SA, Woo HJ, Lee JH, Seo HG (2012). Antioxidative effects of Alisma orientale extract in palmitate-induced cellular injury. Pharm Biol.

[17] Kim EY, Lee JH (2014). The Effect of Alisma orientale Extract on Free Fatty Acid-induced Lipoapoptosis in HepG2 Cells. J Korean Orient Inter Med.

[18] Eun-Kyeong Choi YJC, Hea Jung Y, Ki-Suk K, In-Seung L, Jong-Chan Jang K-HK, Ji Hyun B, Yumi K, Se Hoon K, Young-Hwan Cho YPJ, Na Young Y, Mi-Yeon S, Hyeung-Jin J (2015). Coix seed extract attenuates the high-fat induced mouse obesity via PPARγ and C/EBPα downregulation. Mol Cell Toxicol.

[19] Shin M-H, Choi E-K, Kim K-S, Kim K-H, Jang YP, Ahn KS (2014). Hexane Fractions of Bupleurum falcatum L. Stimulates Glucagon-Like Peptide-1 Secretion through -Mediated Pathway. Evidence-Based Complementary and Alternative Medicine.

[20] Shin M-H, Park Y, Kim K-S, Cho D, Uh I, Kim K-H (2014). The anti-inflammatory effects of Alisma herb extract on allergic asthma mouse model. Mol Cell Toxicol.

[21] Qian Ding JB, Zhao W, Lu J, Zhu H, Chen X (2016). Ethanol enhances cucurbitacin B-induced apoptosis by inhibiting cucurbitacin B-induced autophagy in LO2 hepatocytes. Mol Cell Toxicol.

[22] Mei Nu Cui H-HY, Nan Ee L, Hye Yun K, Chang-Nim I, Yong-Sam K, Jeong-Hwa L (2016). Depletion of BIS sensitizes A549 cells to treatment with cisplatin. Mol Cell Toxicol.

[23] Koh-Woon Kim I-SL, Lee W-J, Park J, Chung WS, Cho J-H, Lee S-L, Jang H-J, Chung S-H (2016). Osteogenic differentiation of human mesenchymal stem cells promoted by the crude extracts of the mixture of Cortex mori radicis, Patrinia saniculaefolia. Mol Cell Toxicol.

[24] Chunfang Zhang DC, Xianbao L, Lili D (2016). Role of brominated diphenyl ether-209 in the proliferation and apoptosis of rat cultured neural stem cells in vitro. Mol Cell Toxicol.

[25] Barnes DG, Vidiassov M, Ruthensteiner B, Fluke CJ, Quayle MR, McHenry CR (2013). Embedding and publishing interactive, 3-dimensional, scientific figures in Portable Document Format (PDF) files. PLoS One.

[26] Wan Y, Liu LY, Hong ZF, Peng J (2014). Ethanol extract of attenuates hepatic lipid accumulation via AMPK activation in human HepG2 cells. Exp Ther Med.

[27] Jang E, Shin MH, Kim KS, Kim Y, Na YC, Woo HJ (2014). Anti-lipoapoptotic effect of Artemisia capillaris extract on free fatty acids-induced HepG2 cells. BMC Complement Altern Med.

[28] Bradbury MW (2006). Lipid metabolism and liver inflammation. I. Hepatic fatty acid uptake: possible role in steatosis. Am J Physiol Gastrointest Liver Physiol.

[29] Xu L, Yu J, Zhai D, Zhang D, Shen W, Bai L (2014). Role of JNK Activation and Mitochondrial Bax Translocation in Allicin-Induced Apoptosis in Human Ovarian Cancer SKOV3 Cells. Evidence-based Complementary and Alternative Medicine.

[30] Watanabe S, Hashimoto E, Ikejima K, Uto H, Ono M, Sumida Y (2015). Evidence-based clinical practice guidelines for nonalcoholic fatty liver disease/nonalcoholic steatohepatitis. J Gastroenterol.

[31] Wensheng L, Baker SS, Baker RD, Zhu L. Antioxidant Mechanisms in Nonalcoholic Fatty Liver Disease. Curr Drug Targets. 2015;16(12):1301–14. 10.2174/1389450116666150427155342 25915484

[32] Anavi S, Ni Z, Tirosh O, Fedorova M (2015). Steatosis-induced proteins adducts with lipid peroxidation products and nuclear electrophilic stress in hepatocytes. Redox Biol.

[33] Ricchi M, Odoardi MR, Carulli L, Anzivino C, Ballestri S, Pinetti A (2009). Differential effect of oleic and palmitic acid on lipid accumulation and apoptosis in cultured hepatocytes. J Gastroenterol Hepatol.

[34] Baylin A, Kabagambe EK, Siles X, Campos H (2002). Adipose tissue biomarkers of fatty acid intake. Am J Clin Nutr.

[35] Yao HR, Liu J, Plumeri D, Cao YB, He T, Lin L (2011). Lipotoxicity in HepG2 cells triggered by free fatty acids. Am J Transl Res.

[36] Donnelly KL, Smith CI, Schwarzenberg SJ, Jessurun J, Boldt MD, Parks EJ (2005). Sources of fatty acids stored in liver and secreted via lipoproteins in patients with nonalcoholic fatty liver disease. J Clin Investig.

[37] Pettinelli P, Obregón AM, Videla LA (2011). Molecular mechanisms of steatosis in nonalcoholic fatty liver disease. Nutr Hosp.

[38] Cazanave SC, Wang X, Zhou H, Rahmani M, Grant S, Durrant DE (2014). Degradation of Keap1 activates BH3-only proteins Bim and PUMA during hepatocyte lipoapoptosis. Cell Death Differ.

[39] Lee SJ, Jung YH, Oh SY, Song EJ, Choi SH, Han HJ (2015). Vibrio vulnificus VvhA induces NF-[kappa]B-dependent mitochondrial cell death via lipid raft-mediated ROS production in intestinal epithelial cells. Cell Death and Disease.

[40] Yamada H, Kikuchi S, Inui T, Takahashi H, Kimura K-I (2014). Gentiolactone, a Secoiridoid Dilactone from *Gentiana triflora*, Inhibits TNF-α, iNOS and Cox-2 mRNA Expression and Blocks NF-kB Promoter Activity in Murine Macrophages. PLoS One.

[41] Brodsky M, Halpert G, Albeck M, Sredni B (2010). The anti-inflammatory effects of the tellurium redox modulating compound, AS101, are associated with regulation of NFkappaB signaling pathway and nitric oxide induction in macrophages. J Inflamm (London, England).

[42] Tsatsanis C, Androulidaki A, Venihaki M, Margioris AN (2006). Signalling networks regulating cyclooxygenase-2. Int J Biochem Cell Biol.

